# Supplementary material for: Serial Electron Diffraction Data Processing With diffractem and CrystFEL
Source: Front Mol Biosci. 2021 May 19;8:624264. doi: 10.3389/fmolb.2021.624264 (PMC8171297; doi:10.3389/fmolb.2021.624264)
Supplement: Supplementary file 2 [file Presentation4.PDF]

In [ ]:
```
%load_ext autoreload
%autoreload 2
```

In [1]:
```
import matplotlib.pyplot as plt
from diffractem import tools, version
from diffractem.dataset import Dataset
from diffractem.stream_parser import StreamParser, augment_stream
from diffractem import pre_proc_opts
import numpy as np
import pandas as pd
import dask.array as da
# from dask.distributed import Client, LocalCluster
import dask
# import h5py

opts = pre_proc_opts.PreProcOpts('preproc.yaml')
cfver = !{opts.im_exc} -v
print(cfver)
```

```
['CrystFEL: 0.9.1+886ae521', 'License GPLv3+: GNU GPL version 3 or later <http://gnu.org/licenses/gpl.html>.', 'This is free soft
ware: you are free to change and redistribute it.', 'There is NO WARRANTY, to the extent permitted by law.', '', 'Written by Thom
as White and others.']
```

Indexing and Integration

...using *CrystFEL's* `indexamajig` tool and several wrappers around it. What you need to begin:

- a virtual-geometry data file, which you should have created during preprocessing. It contains, first and foremost, all necessary information about the beam center and Bragg peak positions.
- a refined unit-cell file, which you can generate using `peak_processing.ipynb`, and good geometry settings in your `.yaml` config file. If unsure about ellipticity, double check using `peak_processing.ipynb`.

First, we define the list of shot list fields which should go into the output stream file of indexing. (See `indexamajig --copy-hdf5-filed`). Here, we only use the really crucial ones, without which the stream file will be hard to use later on.

In [2]:
```
stream_fields = ['frame', 'sample', 'region', 'crystal_id', 'run',
                 '_Event', '_file', 'center_x', 'center_y']

# filter to only take the ones that are actually present
ds_ctr = Dataset.from_files('virtual.h5', open_stacks=True, chunking=-1)
stream_fields = [f'/%/shots/{f}' for f in  stream_fields if f in ds_ctr.shots.columns]

# generate geometry file for virtual geometry from yaml file parameters.
opts.load()
tools.make_geometry(opts, 'virtual.geom', image_name='zero_image', xsize=1024, ysize=1024, mask=False)
```

```
Single-file dataset, disabling parallel I/O.
No feature list in data set ('/%/map/features not found in virtual.h5.'). That's ok if it's a virtual or info file.
Persisting stacks to memory: index, nPeaks, peakTotalIntensity, peakXPosRaw, peakYPosRaw
```

Direct local execution

...generates a shell script `im_run.sh` containing the CrystFEL call, to directly run on this machine, using a number of processes defined in the `procs` argument. All parameters for indexing are set in the `preproc.yaml` file.

In [3]:
```
opts.load() # often reload the opts so they remain updated
tools.call_indexamajig('virtual.lst', 'virtual.geom', script='im_run.sh',
                       output='virtual.stream',  cell='gencell.cell', im_params=opts.indexing_params,
                       copy_fields=stream_fields, procs=40)
```

In [4]:
```
# for the curious cats
!cat im_run.sh
```

```
indexamajig -g virtual.geom -i virtual.lst -o virtual.stream -j 40 -p gencell.cell --indexing=pinkIndexer --integration=rings-nog
rad-nocen --int-radius=3,4,6 --peaks=cxi --hdf5-peaks=/entry/data --no-revalidate --max-res=400 --pinkIndexer-considered-peaks-co
unt=4 --pinkIndexer-angle-resolution=4 --pinkIndexer-refinement-type=5 --pinkIndexer-thread-count=1 --pinkIndexer-tolerance=0.1 -
-pinkIndexer-reflection-radius=0.001 --pinkIndexer-max-resolution-for-indexing=2 --min-peaks=15 --no-refine --no-retry --no-check
-peaks --temp-dir=/scratch/diffractem --copy-hdf5-field=/%/shots/sample --copy-hdf5-field=/%/shots/region --copy-hdf5-field=/%/sh
ots/crystal_id --copy-hdf5-field=/%/shots/run --copy-hdf5-field=/%/shots/_Event --copy-hdf5-field=/%/shots/_file --copy-hdf5-fiel
d=/%/shots/center_x --copy-hdf5-field=/%/shots/center_y
```

Version for clusters

...which splits up the patterns into sections of `shot_per_run`, and generates a script file that submits them independently to a SLURM queue manager. Similar to CrystFEL's `turbo-index-slurm`, but a bit more streamlined. All required files for indexing can be optionally packed into a `.tar.gz` file, which can be uploaded to a cluster right away and run there.

Here, `procs` defines the number of parallel processes with which a chunk of `shots_per_run` shots is processed; additionally `threads` can be defined, which are used by *pinkIndexer*. Vs `procs`, this is especially useful to save memory.

Here it is important, that the `exc` argument gets the path to the `indexamajig` executable on your cluster.

In [5]:
```
tar, script = tools.call_indexamajig_slurm('virtual.lst', 'virtual.geom', name='lyso_idx', cell='refined.cell',
                              im_params=opts.indexing_params, procs=4, threads=2, shots_per_run=50,
                              tar_file='virtual.tar.gz', temp_dir='$TMP_LOCAL', copy_fields=stream_fields,
                              exc='$HOME/SHARED/EDIFF/software/crystfel9/bin/indexamajig',
                                          local_bin_dir='/opts/crystfel_master/bin')
```

```
Wrote self-contained tar file lyso_idx.tar.gz. Upload to your favorite cluster and extract with: tar -xf lyso_idx.tar.gz
Run indexing by calling ./im_run_lyso_idx.sh
```

Template for sending to/receiving from a cluster

In [ ]:
```
# upload immediately to your cluster
# remote = 'rbuecke1@login.gwdg.de:~/SHARED/EDIFF/lyso_redo'
# !ssh {remote.split(":")[0]} 'mkdir -p {remote.split(":")[1]}'
# !scp {tar} {remote}
```

In [ ]:
```
# concat streams on server and transfer back
# name = 'lyso_idx'
# cmd = f'ssh {remote.split(":")[0]} \"cat {remote.split(":")[1]}/partitions/*.stream > {remote.split(":")[1]}/virtual.stream\"'
# !{cmd}
# !scp -r {remote}/virtual.stream .
```

Integration

Now we have the file `virtual.stream`, which contains our indexing solution! We now need to run `indexamajig` a second time, this time on our actual data and using `indexing=file`. This way, instead of running a fresh indexing, it will take a *solution file* (`.sol`), which contains per line:

- The filename and CrystFEL event identifier of an indexed crystal (2 parameters)
- The reciprocal lattice vectors in laboratory frame (9 parameters)
- The shift of the detector for that pattern (2 parameters). This is particularly important, as here we can inject the variable beam center of our datasets, on top of the (much smaller) residual shift that a prediction refinement after index might have found Of course this file is generated automatically.

But first we have to make a geometry file, using our optimized geometry parameters (including ellipticity refinement from `proc_peaks.ipynb`). All required parameters are in `preproc.yaml`.

In [6]:
```
# make the final geometry
opts = pre_proc_opts.PreProcOpts('preproc.yaml')
geo = tools.make_geometry(opts, 'refined.geom', write_mask=True)
```

Solution file from dataset

This is usual the better (if a bit slower version) compared to that belo. Here, a Dataset object is loaded from disk. Now, the stored crystal identification data for each shot in the Dataset (i.e.: `sample`, `region`, `run`, `crystal_id`) are used for matching. You can hence now integrate even from a totally different set of patterns (e.g. a different aggregation range, or even a set with all non-aggregated data - the crystal ID data will just repeat for each frame).

The solution should have the same name as the `.lst` file, which is inherently assumed by the `file` indexer.

In [7]:
```
ds_all = Dataset.from_files('hits-allframe.lst', open_stacks=False)
ds_all.get_indexing_solution('virtual.stream', sol_file='hits-allframe.sol')
```

Out[7]:

	file	Event	astar_x	astar_y	astar_z	bstar_x	bstar_y	bstar_z	cstar_x	cstar_y	csta
**20**	proc_data/LysoS1_001_00000_allframe_hit.h5	entry//20	-0.123163	-0.030406	0.012811	0.024581	-0.116320	-0.043168	0.046089	-0.081634	0.2494
**21**	proc_data/LysoS1_001_00000_allframe_hit.h5	entry//21	-0.123163	-0.030406	0.012811	0.024581	-0.116320	-0.043168	0.046089	-0.081634	0.2494
**22**	proc_data/LysoS1_001_00000_allframe_hit.h5	entry//22	-0.123163	-0.030406	0.012811	0.024581	-0.116320	-0.043168	0.046089	-0.081634	0.2494
**23**	proc_data/LysoS1_001_00000_allframe_hit.h5	entry//23	-0.123163	-0.030406	0.012811	0.024581	-0.116320	-0.043168	0.046089	-0.081634	0.2494
**24**	proc_data/LysoS1_001_00000_allframe_hit.h5	entry//24	-0.123163	-0.030406	0.012811	0.024581	-0.116320	-0.043168	0.046089	-0.081634	0.2494
**...**	...	...	...	...	...	...	...	...	...	...	
**13475**	proc_data/LysoS2_046_00000_allframe_hit.h5	entry//345	-0.034899	-0.121375	0.008912	-0.120469	0.035137	0.014176	-0.030315	-0.009250	-0.2604
**13476**	proc_data/LysoS2_046_00000_allframe_hit.h5	entry//346	-0.034899	-0.121375	0.008912	-0.120469	0.035137	0.014176	-0.030315	-0.009250	-0.2604
**13477**	proc_data/LysoS2_046_00000_allframe_hit.h5	entry//347	-0.034899	-0.121375	0.008912	-0.120469	0.035137	0.014176	-0.030315	-0.009250	-0.2604
**13478**	proc_data/LysoS2_046_00000_allframe_hit.h5	entry//348	-0.034899	-0.121375	0.008912	-0.120469	0.035137	0.014176	-0.030315	-0.009250	-0.2604
**13479**	proc_data/LysoS2_046_00000_allframe_hit.h5	entry//349	-0.034899	-0.121375	0.008912	-0.120469	0.035137	0.014176	-0.030315	-0.009250	-0.2604

11770 rows × 13 columns

Solution file directly from stream

Another option to get a `.sol` file is to run the `stream2sol` command-line tool. While fast, this is restricted to the case that you want to integrate from the exact same images as those you used for indexing (i.e., the ones you used to create `virtual.h5` in `preprocessing.ipynb`, and you have fields with mm-calibrated shifts in your stream (might not be the case).

In [ ]:
```
cmd = tools.make_command('stream2sol', input='virtual.stream', output='hits_agg.sol',
                  event_field='hdf5/%/shots/_Event', file_field='hdf5/%/shots/_file',
                  x_shift_field='hdf5/%/shots/shift_x_mm', y_shift_field='hdf5/%/shots/shift_y_mm')
print('Running conversion command:', cmd)
!{cmd};
```

Run the integration

Now we're all set to integrate the data set. The parameters for integration are all set in the `integration_params` structure in `preproc.yaml`. It can be well worth playing with them, especially `int-radius` and `integration`. For the latter, we recommend to stick to `rings-nograd-nocen`, if your patterns are background-subtracted. Otherwise `rings-grad-nocen` might work better. Abstain from anything with `cen` in it, as it will strongly bias high-resolution peak values. `Overpredict` might help if you plan to do merging with partiality correction (though it doesn't much in our experience), but absolutely don't do it for Monte-Carlo merging.

Always keep `no-revalidate`, `no-retry`, `no-refine`, `no-check-cell` active.

After you've run the command (might take a fair bit), you'll have a stream file ready for merging. See `merging.ipynb`.

In [8]:
```
stream_name = f'streams/hits-allframe.stream'
list_file = 'hits-allframe.lst'
copy_fields = ['sample', 'region', 'crystal_id', 'run',
               'adf1', 'adf2', 'lor_hwhm', 'center_x', 'center_y']
tmp_dir = '/scratch/diffractem' # set to '.' if you want to stay here

opts.load()

%cp {list_file.rsplit('.', 1)[0]}.sol {tmp_dir}

copy_fields = [f'/%/shots/{cf}' for cf in copy_fields]
cfcall = tools.call_indexamajig(list_file, 'refined.geom',
                                output=stream_name,
                                cell='refined.cell',
                                im_params=opts.integration_params,
                                procs=40, exc='/opts/crystfel_hash/bin/indexamajig',
                                copy_fields=copy_fields, temp_dir=tmp_dir)

print('--- RUN THIS ---------------')
print(cfcall)
```

```
--- RUN THIS ---------------
/opts/crystfel_hash/bin/indexamajig -g refined.geom -i hits-allframe.lst -o streams/hits-allframe.stream -j 40 -p refined.cell --
indexing=file --peaks=cxi --hdf5-peaks=/entry/data --no-revalidate --int-radius=3,4,6 --integration=rings-nograd-nocen --no-retry
--no-refine --no-check-cell --temp-dir=/scratch/diffractem --copy-hdf5-field=/%/shots/sample --copy-hdf5-field=/%/shots/region --
copy-hdf5-field=/%/shots/crystal_id --copy-hdf5-field=/%/shots/run --copy-hdf5-field=/%/shots/adf1 --copy-hdf5-field=/%/shots/adf
2 --copy-hdf5-field=/%/shots/lor_hwhm --copy-hdf5-field=/%/shots/center_x --copy-hdf5-field=/%/shots/center_y
```
