# Supplementary material for: Serial Electron Diffraction Data Processing With diffractem and CrystFEL
Source: Front Mol Biosci. 2021 May 19;8:624264. doi: 10.3389/fmolb.2021.624264 (PMC8171297; doi:10.3389/fmolb.2021.624264)
Supplement: Supplementary file 4 [file Presentation3.PDF]

```
In [ ]:
# useful for development work
%load_ext autoreload
%autoreload 2
```

```
In [ ]:
import hdf5plugin # required to access LZ4-encoded HDF5 data sets
import matplotlib.pyplot as plt
from diffractem import tools, proc_peaks, version, pre_proc_opts
from diffractem.dataset import Dataset
import numpy as np
import os
import subprocess
import io
from scipy.optimize import least_squares, minimize
from numpy import fft
import dask as da

#%matplotlib widget
```

Cell and geometry refinement from Bragg peaks

This notebook exclusively acts on the Bragg peaks found during preprocessing, and mostly uses the functionality provided by `diffractem.proc_peaks`. Currently, this comprises:

- Refinement elliptic distortion of the patterns. Note that distortion correction does *not* require a calibration sample, it can be done using the data itself. If the d-spacings are known (or some of them, at least), the camera length can be calibrated, too.
- Computation of a virtual powder pattern, and the distribution of peak distances from all diffraction patterns. The latter is most useful to determine the cell parameters, as the lengths of the primitive unit cell should clearly show up at low resolution.
- Auto-Refinement of the unit cell using the previously computed powder and distances patterns. The final cell can be exported in CrystFEL format.

First, load a data set and get all peak-related data. An `image_info.h5`-type file as is created by `proc2d.get_pattern_info` serves the purpose perfectly.

```
In [2]:
ds = Dataset.from_files('image_info.h5', chunking=-1)
pkd = da.compute({k: v for k, v in ds.stacks.items() if 'peak' in k.lower()})[0]
```

```
Single-file dataset, disabling parallel I/O.
No feature list in data set ('/%/map/features not found in image_info.h5.'). That's ok if it's a virtual or info file.
Persisting stacks to memory: nPeaks, peakTotalIntensity, peakXPosRaw, peakYPosRaw
```

Ellipticity refinement

Residual elliptical distortions of the pattern are inevitable in a TEM and significantly impact the quality of indexing and integration. Luckily, they can be accounted for easily. Their values are stored in the configuration `.yaml` file. For a first check, just run this cell and modify the values initially loaded from the file to your liking. The analysis is then done on a 2D histogram of peak positions in polar coordinates (radius, azimuth), that is an azimuthally resolved ("segmented") powder pattern. Adjust `rad_range` such that you have a couple of strong lines visible.

If those lines are rather waves, your ellipticity is not well corrected. To get close to the proper values, play with the parameters in `preproc.yaml` until you smooth them out. On the right panel you see the square of the mean pattern, and the product of the ring pattern with itself rotated by 90°. Make those two curves match as closely as possible. The two values in the caption also give a good indication: make the ellipticity variance small, and the quadrant correlation large. And have fun!

Spoiler: in this data set, the ratio is 1.023 and the angle 83°.

```
In [14]:
# ellipticity checker
opts = pre_proc_opts.PreProcOpts('preproc.yaml')

# radial range to show (in pixels)
rad_range = (10, 80)

peakdata = proc_peaks.get_pk_data(pkd['nPeaks'], pkd['peakXPosRaw'], pkd['peakYPosRaw'],
                                  ds.shots.center_x.values,
                                  ds.shots.center_y.values,
                                  opts=opts,
                                  return_vec=False)

az = np.arctan2((opts.y_scale*peakdata['peakYPosCor']), peakdata['peakXPosCor']).ravel()
tt = (((opts.y_scale*peakdata['peakYPosCor'])**2 + peakdata['peakXPosCor']**2)**.5).ravel()

# powder pattern in polar coordinates
powder_polar = np.histogram2d(tt, az*180/np.pi,
                              bins=[np.linspace(*rad_range, 200), np.linspace(-180, 180, 20)])
corr = powder_polar[0] * np.roll(powder_polar[0], powder_polar[0].shape[1]//4, axis=1)

plt.close('all')
fh, ax = plt.subplots(1, 2, figsize=(8,5), sharey=True)
ax[0].pcolormesh(powder_polar[2][:-1], powder_polar[1][:-1], powder_polar[0])
ax[0].set_xlabel('Azimuth (deg)')
ax[0].set_ylabel('Corrected radius')
ax[1].plot(np.nanmean(powder_polar[0]**2, axis=1), powder_polar[1][:-1], label='Mean squared pattern')
ax[1].plot(np.nanmean(corr, axis=1), powder_polar[1][:-1], label='Quadrant correlation')
ax[1].legend()
ax[1].set_xlabel('Mean squared counts')

plt.title(f'Median ellipticity variance: {np.nanmedian((np.nanvar(powder_polar[0], axis=1)/np.nanmean(powder_polar[0],axis=1))):.2f}'
        f'Rel. quadrant correlation: {np.mean(corr)/np.mean(powder_polar[0]**2):.3g}')
```

```
/opts/anaconda/envs/preproc/lib/python3.7/site-packages/ipykernel_launcher.py:36: RuntimeWarning: invalid value encountered in true_d
ivide
```

```
Out[14]: Text(0.5, 1.0, 'Median ellipticity variance: 0.94 \nRel. quadrant correlation: 0.82')
```

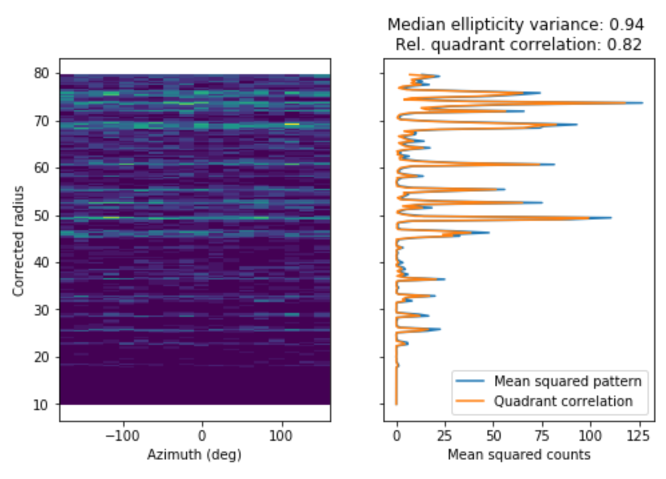

Ellipticity grid search

Once you're close, but not quite sure, you can run a grid search over angles and ellipticity to find the optimum. The correlations are computed in parallel for performance.

```
In [15]:
angles = np.arange(80, 90, 1)
ratio = np.arange(1.02, 1.03, 0.0005)

def cost(p):
    peakdata = proc_peaks.get_pk_data(pkd['nPeaks'], pkd['peakXPosRaw'], pkd['peakYPosRaw'],
                                  ds.shots.center_x.values,
                                  ds.shots.center_y.values,
                                  opts=opts,
                                  return_vec=False,
                                      el_rat=p[0], el_ang=p[1])

    az = np.arctan2((opts.y_scale*peakdata['peakYPosCor']), peakdata['peakXPosCor']).ravel()
    tt = (((opts.y_scale*peakdata['peakYPosCor'])**2 + peakdata['peakXPosCor']**2)**.5).ravel()
    powder_polar = np.histogram2d(tt, az*180/np.pi,
                                  bins=[np.linspace(*rad_range, 200), np.linspace(-180, 180, 20)])

    return np.mean(powder_polar[0]**2)/np.mean(powder_polar[0] * np.roll(powder_polar[0], powder_polar[0].shape[1]//4, axis=1)) - 1

from concurrent.futures import ProcessPoolExecutor
X, Y = np.meshgrid(ratio, angles)
with ProcessPoolExecutor() as exc:
    foms = exc.map(cost, zip(X.ravel(), Y.ravel()))
foms = np.array(list(foms)).reshape(X.shape)

# show result
plt.figure()
plt.pcolormesh(ratio, angles, foms)
plt.colorbar()
plt.ylabel('Elliptical axis')
plt.xlabel('Ellipticity')
plt.title('Ellipticity correction')
```

```
Out[15]: Text(0.5, 1.0, 'Ellipticity correction')
```

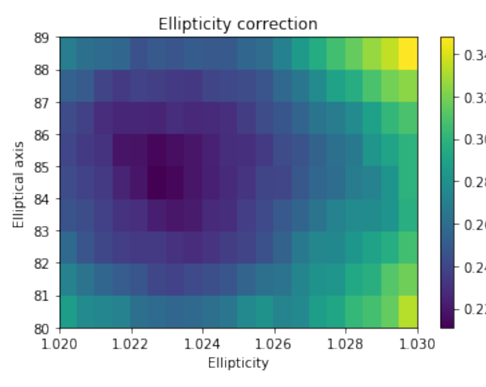

Finalize

Please make sure to enter the found optimal ellipticity into your `.yaml` file in any case!

Virtual Powder Pattern

A quick look, with and without intensity scaling - just to get a feeling. It should look nice and crisp. If it does, proceed!

```
In [16]:
opts.load()
plt.figure()
peakdata = proc_peaks.get_pk_data(pkd['nPeaks'], pkd['peakXPosRaw'], pkd['peakYPosRaw'],
                                  ds.shots.center_x.values,
                                  ds.shots.center_y.values, pk_I=pkd['peakTotalIntensity'],
                                  opts=opts, return_vec=True)
powder, svec = np.histogram(10/peakdata['peakD'].ravel(), bins=np.linspace(0.3,3,1000))
plt.plot(svec[:-1], powder, 'k');
plt.xlabel('Scattering vector (1/nm)')
plt.ylabel('Frequency')
ax2 = plt.twinx()
powder, svec = np.histogram(10/peakdata['peakD'].ravel(), bins=np.linspace(0.3,3,1000),
                            weights=peakdata['peakTotalIntensity'].ravel(), density=True)
ax2.plot(svec[:-1], powder, 'r')
ax2.set_ylabel('Rel. Intensity')
ax2.yaxis.label.set_color('r')
plt.title('Scattering vector distribution');
```

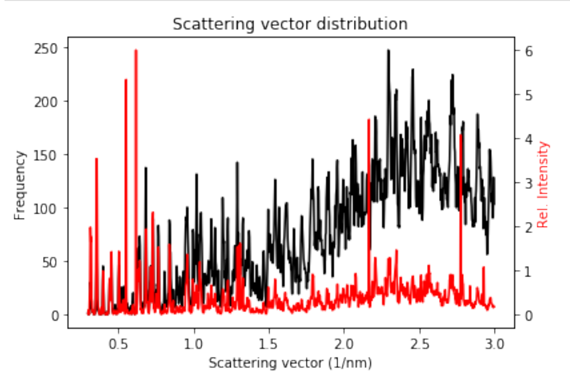

Generate peak pair distribution

Now, for each pattern, the autocorrelation function of peak positions in each diffraction pattern is computed:

$$\mathrm{ACF}(\Delta x, \Delta y) = \sum_i \sum_j w_{ij} \delta(x_i - x_j, y_i - y_j),$$

where (x_i, y_i) are the positions of the found peaks, $w_{ij} = 1$ for `I=None` and $w_{ij} = I_i \cdot I_j$ if peak intensities are provided as argument. Ther result will have strong peaks around typical peak distances, that, especially for near-zone-axis patterns, correspond to low-lying d-spacings, down to the primitive unit cell lengths.

Both 2D autocorrelations (with peak distance vectors) and their radial projection (just containing peak distances) are computed. The former could be used as input for an EDIFF-type cell finding algorithm [Jiang et al. 2009, doi:10.1107/S0907444909003163], whereas the latter is used for unit-cell refinement.

Finally a plot is shown containing the found radial distribution and the virtual powder. If everything goes well, there should be strong coinciding peaks. However the pair distance distribution should have some very strong peaks at low resolutions that are absent from the normal powder pattern. Those will mostly contribute to refinement below.

Mind that this computation can take a while. Important parameters are `oversample` - which defines how precisely the actual peak positions are used (i.e., how "super-resolution" the result will be), and the minimum d-spacing that you're interested in (set by `d_min`)

```
In [17]:
d_min = 4
oversample = 4
shot_selection = peakdata['nPeaks'] > 20

out_rad = int((opts.wavelength / d_min) / (opts.pixel_size / opts.cam_length)) + 1
acfs, r_acfs = proc_peaks.get_acf(peakdata['nPeaks'][shot_selection],
                                  peakdata['peakXPosCor'][shot_selection,:],
                                  peakdata['peakYPosCor'][shot_selection,:],
                                  I = None,
                                  output_radius=out_rad, roi_length=512,
                                  oversample=oversample)

# scattering vector axis for distances
s_d = np.arange(r_acfs.shape[1]) * opts.pixel_size/opts.cam_length/(.1*opts.wavelength)/ovrs

fh, axh = plt.subplots(2,1, figsize=(8,5), sharex=True)
powder, svec = np.histogram(10/peakdata['peakD'].ravel(), bins=np.linspace(0.3,s_d.max(),len(s_d)))
axh[0].plot(svec[1:]/2 + svec[:-1]/2, powder, color='b')
axh[0].legend(['Peak resolution distribution'])
axh[0].grid(True)
axh[1].plot(s_d,r_acfs.mean(axis=0), color='r')
axh[1].set_xlabel('Scattering vector (1/nm)')
axh[1].legend(['Peak pair distance distribution'])
axh[1].grid(True)
axh[0].set_title('Virtual powder')
```

```
Out[17]: Text(0.5, 1.0, 'Virtual powder')
```

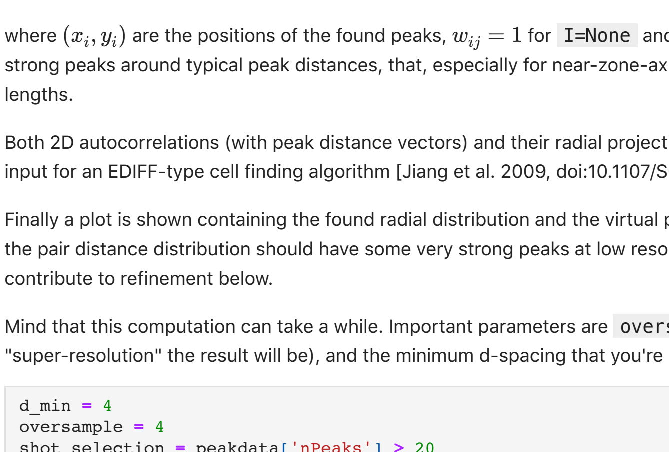

Refine unit cell

...from distance distribution and/or virtual powder. Here it's done in two steps: first using the distances (which are sensitive to the primitive cell lengths and immediately should snap on them), then using the virtual powder (which might be a bit sharper especially at high resolutions). Finally, the refined unit cell is exported to a CrystFEL cell file. All is done using a `proc_peaks.Cell` object, which provides a `refine_powder` method that does it pretty much automatically.

In practice, it might be good enough to just use the distances (or the powder, if you know you're already close).

Note that if you find that all lengths proportionally deviate from your expectation, it's not unlikely that your camera length is off.

```
In [18]:
# refine peaks using cross-correlation, derivative, or distance metric
# set initial cell
C0 = proc_peaks.Cell.tetragonal(78, 38, 'P')

# define minimum d-spacings for the refinement
dmin_d = 13 # for distances
dmin_p = 10 # for powder

# get powder pattern. Tweak histogram bins such that it looks smooth but detailed.
powder, s_p = np.histogram(10/peakdata['peakD'].ravel(), bins=np.linspace(0.3,10/dmin_p,300), density=True)
s_p = s_p[1:]/2 + s_p[:-1]/2

# get average distance distribution
distance = r_acfs.mean(axis=0)

C0.init_hkl(dmin_d)
C_d, par_d = C0.refine_powder(s_d, distance, method='distance', min_prom=0.2, length_bound=2)
print('Initial cell: ', C0)
print(f'Distance refinement: {par_d["lsq_result"].x.round(2)}: {par_d["initial_cost"]:.2g} -> {par_d["lsq_result"].cost:.2g}')

C_d.init_hkl(dmin_p)
C_p, par_p = C_d.refine_powder(s_p, powder, method='distance', min_prom=0.4, length_bound=2)
print(f'Powder refinement: {par_p["lsq_result"].x.round(2)}: {par_p["initial_cost"]:.2g} -> {par_p["lsq_result"].cost:.2g}')

# make a plot for powder-refinement results
plt.close('all')
fh, axh = plt.subplots(1,1, figsize=(8,5), sharex=True)
plt.vlines(10/C0.d(unique=True), 0, powder.max(), color='g', alpha=0.2)
plt.vlines(10/C_d.d(unique=True), 0, powder.max(), color='r', alpha=0.2)
plt.vlines(10/C_p.d(unique=True), 0, powder.max(), color='b', alpha=0.2)
plt.plot(s_p, powder, color='b')
plt.plot(par_p['peak_position'], par_p['peak_height'], 'bx')
ax2 = axh.twinx()
ax2.plot(s_d,distance, color='r')
ax2.plot(par_d['peak_position'], par_d['peak_height'], 'rx')
plt.xlim(0, max(10/dmin_d, 10/dmin_p))
plt.xlabel('Scattering vector (1/nm)')
```

```
Initial cell:  Primitive tetragonal cell (unique c) a=78.0000 b=78.0000 c=38.0000 alpha=90.000 beta=90.000 gamma=90.000
Distance refinement: [78.96 37.9 ]: 1.3 -> 0.18
Powder refinement: [79.01 37.94]: 0.34 -> 0.31
```

```
Out[18]: Text(0.5, 0, 'Scattering vector (1/nm)')
```

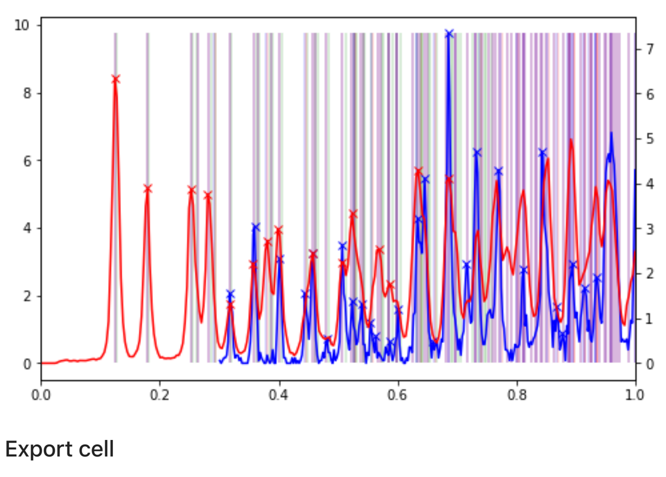

Export cell

Finally, export the refined cell in CrystFEL format. You're now well set up for indexing. See `indexing.ipynb`.

```
In [19]:
C_p.export('refined.cell')
```
