# Supplementary material for: Serial Electron Diffraction Data Processing With diffractem and CrystFEL
Source: Front Mol Biosci. 2021 May 19;8:624264. doi: 10.3389/fmolb.2021.624264 (PMC8171297; doi:10.3389/fmolb.2021.624264)
Supplement: Supplementary file 1 [file Presentation1.PDF]

In [ ]:

```
# for developers
%load_ext autoreload
%autoreload 2
```

In [2]:

```
# import hdf5plugin # required to access LZ4-encoded HDF5 data sets, if not on your global path
import matplotlib.pyplot as plt
from diffractem import version, proc2d, pre_proc_opts, io
from diffractem.dataset import Dataset
from tifffile import imread
import numpy as np
from dask.distributed import Client, LocalCluster, TimeoutError
import os
import pandas as pd

opts = pre_proc_opts.PreProcOpts('preproc.yaml')
# opts.im_exc = 'indexamajig'
cfver = !{opts.im_exc} -v
print(f'Running on diffractem:', version())
print(f'Running on', cfver[0])
print(f'Current path is:', os.getcwd())

pxmask=imread(opts.pxmask)
reference=imread(opts.reference)
```

```
Running on diffractem: v0.3.2-60-g604d443
Running on CrystFEL: 0.9.1+886ae521
Current path is: /localdata/serialed/lys_2019_redo
```

# Pre-processing of SerialED data

...from raw diffraction data in *NeXus* format files to cleaned, sorted, selected, and corrected diffraction data, and accurate information about peak and pattern center positions for further steps. This comprises:

- Aggregation of dose fractionation movies
- Center and peak finding in diffraction patterns
- Hit selection
- Export of peak data files for indexing
- Flat-field, dead-pixel and saturation correction; optionally background subtraction
- Broadcasting of results to single fractionation frames, and different arbitrary cumulations

The central tool are `diffractem.Dataset` objects, which handle file I/O, metadata, subset selection, fast computations, and much more. Also, the image processing functions in `diffractem.proc2d` are essential. By internally using the *dask* framework, Datasets can be larger than memory, computations are lazy (i.e., executed only just when needed) and done in parallel, mostly scaling directly with the number of available cores.

## Initialize *dask.distributed* cluster

This initializes the computation backend. If there is no dask.distributed scheduler running at the specified port (usually 8786), a new one will be created. Make sure that the number of workers and threads make sense. It's a good idea to set it to your workstation's CPU configuration, and explicitly set a fast scratch drive as `local_directory`. In the output of the cell you will find the link for the dashboard of the scheduler, where you can follow the computation progress (NB: if you're connecting to Jupyter through a SSH tunnel, you'll need to open an additional tunnel for the port of the dashboard).

In [3]:

```
cluster_port = 8786

try:
    client = Client(address=f'127.0.0.1:{cluster_port}', timeout='2s')
    print('Running cluster scheduler found and connected.')
    client.run(os.chdir, os.getcwd()); # change the cluster to the current directory
except (OSError, TimeoutError):
    print('Seems no cluster scheduler is running. Starting one.')
    cluster = LocalCluster(host=f'127.0.0.1:{cluster_port}', n_workers=20, threads_per_worker=2,
                    local_directory='/scratch/distributed')
    client = Client(address=f'127.0.0.1:{cluster_port}')

client
```

```
Seems no cluster scheduler is running. Starting one.
```

Out[3]:

| Client | Cluster |
|---|---|
| **Scheduler:** tcp://127.0.0.1:8786 | **Workers:** 20 |
| **Dashboard:** http://127.0.0.1:8787/status | **Cores:** 40 |
| | **Memory:** 270.35 GB |

## Load the raw data set

While the files could just be loaded using a wildcard expression in `Dataset.from_files`, it's a good idea to first get an explicit file list using `io.expand_files`, which you can filter in case you find that some files are troublemakers. This list can explicitly be used as input to `Dataset.from_files`.

An import aspect now is the *chunking* of the Dataset, which defines the size of blocks of the dataset that are loaded into memory and processed separately and in parallel. If you did not use dose fractionation, a value around 20 is a good idea. If you did use dose fractionation, it should be larger, and a multiple of the number of frames per crystal. Read more about chunks in the *dask* documentation.

In this data set we had 25 frames per movie, so we pick `chunking=50`.

In [7]:

```
opts.load() # re-load parameters from the .yaml file

raw_files = io.expand_files('raw_data/*.nxs', validate=True)
print(f'Found {len(raw_files)} raw files. Have fun pre-processing!')
ds = Dataset.from_files(raw_files, chunking=50, )
ds
```

```
Found 34 raw files. Have fun pre-processing!
Persisting stacks to memory:
```

Out[7]:

```
diffractem Dataset object spanning 34 NeXus/HDF5 files
-----
55800 shots (55800 selected)
0 peaks, 0 predictions, 2247 features
1 data stacks: raw_counts
Diffraction data stack: raw_counts
Data files open: True
Data files writable: False
```

### Merge global metadata into shot list

Here, we only merge the shutter time of each movie frame (as they varied between the regions in this set)

In [8]:

```
ds.merge_meta('/%/instrument/detector/collection/shutter_time')
```

## Aggregation/selection

Now, shots that correspond to auxiliary scan points have to be rejected (or based on other criteria in the shot list), and, if dose fractionation was used, movies should be summed over some reasonable range of frames before further processing. (In the final steps, we can work on the separate frames again.)

Now is a good time to have a first look at the (aggregated) dataset using `tools.viewing_widget`. Note that no data is written to disk yet, all operations are done lazily, i.e. just computed in real time when you need it.

In [9]:

```
ds_agg = ds.aggregate(query='frame >= 1 and frame <= 4 and shutter_time == 2',
                      by=['sample', 'region', 'run', 'crystal_id'], how='sum', new_folder='proc_data')
print(f'Have {len(ds_agg.shots)} shots for processing.')
```

```
Monotonous aggregation: True
File/subset remixing: False
Frame aggregation: True
Acq. run aggregation: False
Discarding shot table columns: ['Event', 'frame', 'shot_in_subset']
Persisting stacks to memory:
Have 2146 shots for processing.
```

### Have a first look

Fire up the viewing widget, with some reasonable default parameters. Log is helpful for unprocessed sets.

In [10]:

```
%matplotlib widget
ds_agg.view(Imax=4000, log=True)
```

```
/opts/dev/diffractem/diffractem/tools.py:734: RuntimeWarning: divide by zero encountered in log10
  ih.set_data(np.log10(img_stack[shot,...].compute(scheduler='single-threaded')))
```

```
sample: Lyso190304
region: 12
run: 0
crystal_id: 83
crystal_x: 853.9447674418604
crystal_y: 992.8255813953489
pos_x: 837.3447674418604
pos_y: 993.9751937984497
selected: True
subset: entry
shutter_time: 2.0
agg_len: 4
file:
proc_data/LysoS1_012_00000_agg.h5
file_raw:
raw_data/LysoS1_012_00000.nxs
Event: entry//82
shot_in_subset: 82
Event_raw: entry//2104
```

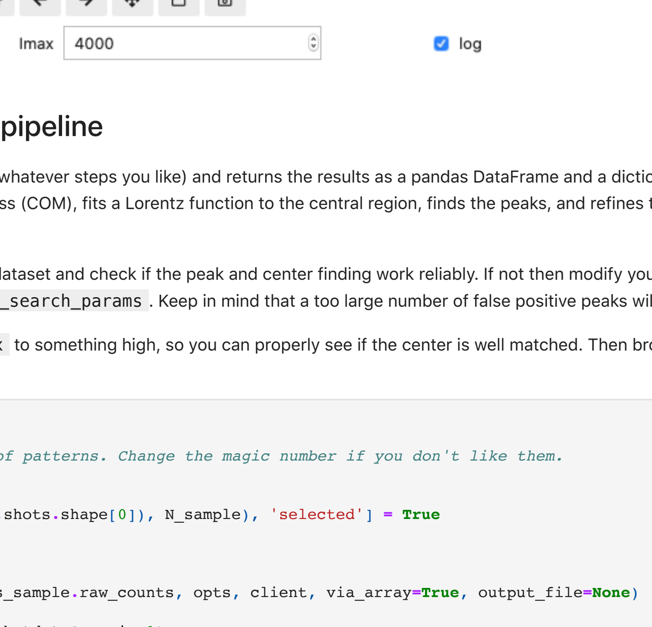

zoom rect, x=1045.4 y=313.838 [0.477]

shot 366 | selected | 2146 of 2146 shots selected. | Imax 4000 | log

## Optimize the peak & center finding pipeline

`get_pattern_info` analyzes diffraction patterns (with whatever steps you like) and returns the results as a pandas DataFrame and a dictionary containing found peak positions in CXI formats. For now, it finds the center of mass (COM), fits a Lorentz function to the central region, finds the peaks, and refines the center position using Friedel mate matching.

Here, you can test this pipeline on a small subset of your dataset and check if the peak and center finding work reliably. If not then modify your `preproc.yaml` file parameters, especially those for peak finding under `peak_search_params`. Keep in mind that a too large number of false positive peaks will confuse the indexer horribly.

For displaying, consider the `log` checkbox and set `Imax` to something high, so you can properly see if the center is well matched. Then browse a bit through the shots and check the quality of the image annotations.

In [11]:

```
N_sample = 30  # Number of sample shots
opts.load() # load changes of YAML file
np.random.seed(4200) # seed the random choice of patterns. Change the magic number if you don't like them.

ds_agg.shots['selected'] = False
ds_agg.shots.loc[np.random.choice(range(ds_agg.shots.shape[0]), N_sample), 'selected'] = True
ds_sample = ds_agg.get_selection()
ds_agg.shots['selected'] = True

shotdata, peakinfo = proc2d.get_pattern_info(ds_sample.raw_counts, opts, client, via_array=True, output_file=None)

ds_sample.shots = pd.concat([ds_sample.shots, shotdata], axis=1)
for k, v in peakinfo.items():
    ds_sample.add_stack(k, v, overwrite=True, persist=True)
```

```
Persisting stacks to memory:
```

In [17]:

```
ds_sample.view(Imax=4000, log=True)
```

```
/opts/dev/diffractem/diffractem/tools.py:734: RuntimeWarning: divide by zero encountered in log10
  ih.set_data(np.log10(img_stack[shot,...].compute(scheduler='single-threaded')))
```

```
sample: Lyso190304
region: 14
run: 0
crystal_id: 14
crystal_x: 540.0980392156863
crystal_y: 156.9281045751634
pos_x: 523.4980392156863
pos_y: 159.41844605159451
selected: True
subset: entry
shutter_time: 2.0
agg_len: 4
file_raw:
raw_data/LysoS1_014_00000.nxs
Event_raw: entry//312
file:
proc_data/LysoS1_014_00000_agg_sel.h
5
Event: entry//0
shot_in_subset: 0
adf1: 31.260875415081845
adf2: 11.972472026020348
center_refine_score:
0.048638357170224l7
center_x: 739.7617520555066
center_y: 283.61060268177096
com_x: 739.1048407182732
com_y: 283.32960959917773
lor_hwhm: 9.468556369077435
lor_pk: 1484.4431114564893
lor_x: 739.3620847791043
lor_y: 283.9966759467171
num_peaks: 66
shift_x_mm: -40.68689636305286
shift_y_mm: -15.598583147497402
```

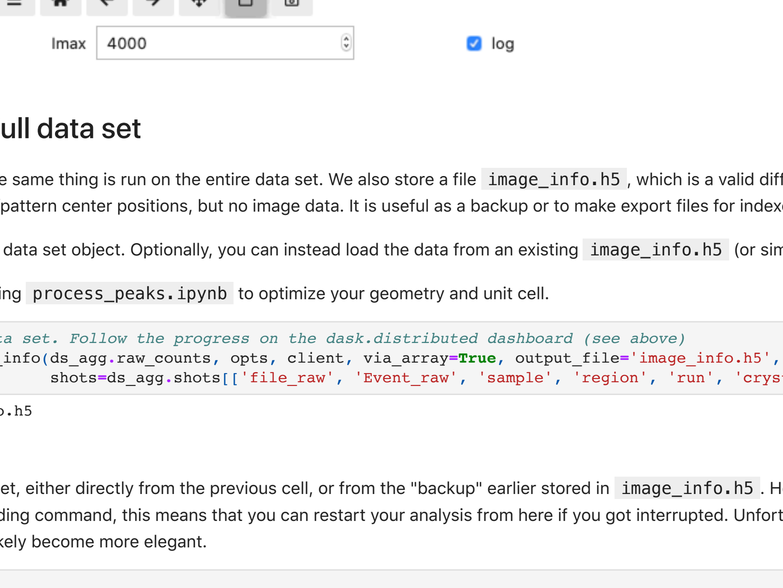

zoom rect, x=702.073 y=507.782 [0.477]

shot 5 | selected | 30 of 30 shots selected. | Imax 4000 | log

## Run center & peak finding on full data set

Now that you've found the optimal parameters, the same thing is run on the entire data set. We also store a file `image_info.h5`, which is a valid diffractem-type (NeXus) HDF5 file, which contains the metadata and peak/pattern center positions, but no image data. It is useful as a backup or to make export files for indexers.

In the second cell, the results are weaved into our data set object. Optionally, you can instead load the data from an existing `image_info.h5` (or similar) file.

Note also, that from this point on you can start using `process_peaks.ipynb` to optimize your geometry and unit cell.

In [14]:

```
# now do the same thing on the whole data set. Follow the progress on the dask.distributed dashboard (see above)
shotdata, peakinfo = proc2d.get_pattern_info(ds_agg.raw_counts, opts, client, via_array=True, output_file='image_info.h5',
                                             shots=ds_agg.shots[['file_raw', 'Event_raw', 'sample', 'region', 'run', 'crystal_id']])
```

```
Wrote analysis results to file image_info.h5
```

### Merging into dataset

We now have the info we want for the entire dataset, either directly from the previous cell, or from the "backup" earlier stored in `image_info.h5`. Here, this is merged into the `Dataset`. If you uncomment the dataset loading command, this means that you can restart your analysis from here if you got interrupted. Unfortunately, this still has to be done using manual *pandas* calls, but this will likely become more elegant.

In [15]:

```
# if you want to restart from here...
# ds_hit = Dataset.from_files('hits.lst', chunking=50)

# merge found shot data into dataset
from_backup = False

if from_backup:
    ds_info = Dataset.from_files('image_info.h5', chunking=-1)
    shots = ds_agg.shots.merge(ds_info.shots, on=['file_raw', 'Event_raw', 'sample', 'region', 'run', 'crystal_id'],
                validate='1:1', how='left', suffixes=('', '_INFO'))
    ds_agg.shots = shots.drop(columns=[c for c in shots.columns if c.endswith('_INFO')])
    for k, v in ds_info.stacks.items():
        ds_agg.add_stack(k, v.rechunk(chunks=(ds_agg.zchunks,) + v.shape[1:]), overwrite=True, persist=True) # rechunk is temporary workaround

else:
    ds_agg.shots = pd.concat([ds_agg.shots.drop(columns=[c for c in shotdata.columns if c in ds_agg.shots.columns]), shotdata], axis=1)
    for k, v in peakinfo.items():
        ds_agg.add_stack(k, v, overwrite=True, persist=True)
```

## Hit selection

define a criterion to select hits, accordsing to the `selection` query string, then show random sample images to get an idea if it made sense. It often makes sense to not only look at the total `num_peaks`, but also `num_lores_peaks` and `frac_lores_peaks`. The resolution limit for what is considered lores is defined as `lores_limit` in inverse nanometres.

Afterwards, you can look (again) at your hit-selected data set `ds_hit` to see if it contains hits only.

In [16]:

```
plt.close('all')
selection = 'num_peaks > 15'

ds_agg.shots['hit'] = ds_agg.shots.eval(selection)
ds_hit = ds_agg.get_selection('hit', file_suffix='_hit.h5')

ds_hit.view(Imax=50)
```

```
1322 shots out of 2146 selected.
Persisting stacks to memory: nPeaks, peakTotalIntensity, peakXPosRaw, peakYPosRaw
```

```
sample: Lyso190304
region: 38
run: 0
crystal_id: 11
crystal_x: 639.9937351943968
crystal_y: 62.00722425598677
pos_x: 626.4937351943968
pos_y: 61.12746806809861
selected: True
subset: entry
shutter_time: 2.0
agg_len: 4
file_raw:
raw_data/LysoS2_038_00000.nxs
Event_raw: entry//281
adf1: 30.705749898806744
adf2: 11.09915803364941
center_refine_score:
0.018659374115855852
center_x: 746.4798779891358
center_y: 281.6398570910109
com_x: 746.2666939569559
com_y: 282.23286822660424
lor_hwhm: 9.48781078355626
lor_pk: 1488.243093805072
lor_x: 746.2591773000288
lor_y: 282.8308108369381
num_peaks: 39
shift_x_mm: -41.05639328940247
shift_y_mm: -15.4901921400056
hit: True
file:
proc_data/LysoS2_038_00000_agg_hit.h
5
Event: entry//11
shot_in_subset: 11
```

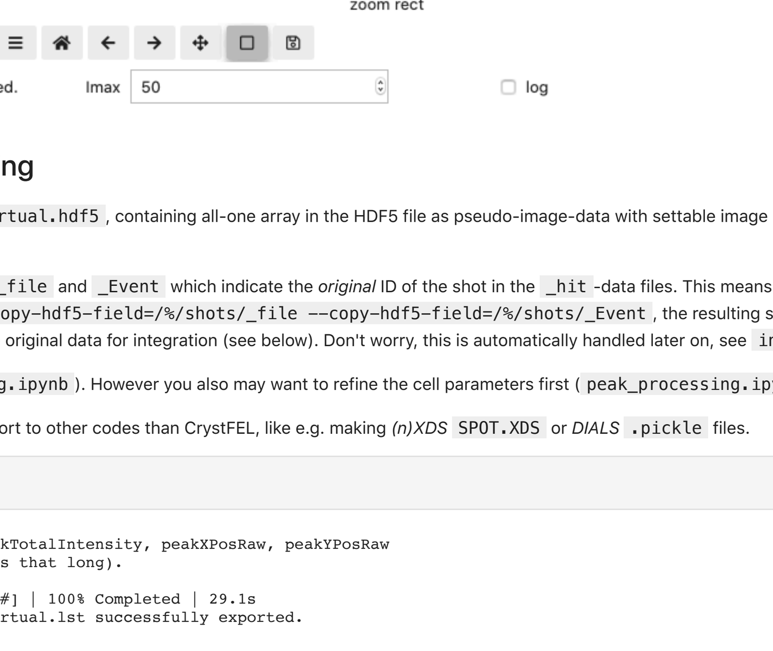

zoom rect

shot 917 | selected | 1322 of 1322 shots selected. | Imax 50 | log

## Creation of peak file for indexing

...based on the hits data set. Generates a file `virtual.hdf5`, containing all-one array in the HDF5 file as pseudo-image-data with settable image size, but keeps the peak positions, referenced to the image center.

Also the shot table is kept, with added columns `_file` and `_Event` which indicate the *original* ID of the shot in the `_hit`-data files. This means that when running `indexamajig` with corresponding options `--copy-hdf5-field=/%/shots/_file --copy-hdf5-field=/%/shots/_Event`, the resulting stream file contains the original shot ID can easily be mapped back to the original data for integration (see below). Don't worry, this is automatically handled later on, see `indexing.ipynb`.

After this step, you can start indexing (`indexing.ipynb`). However you also may want to refine the cell parameters first (`peak_processing.ipynb`).

As a TODO: this is the point where one could export to other codes than CrystFEL, like e.g. making *(n)XDS* `SPOT.XDS` or *DIALS* `.pickle` files.

In [13]:

```
%rm virtual.h5
ds_hit.write_virtual_file()
```

```
1322 shots out of 1322 selected.
Persisting stacks to memory: nPeaks, peakTotalIntensity, peakXPosRaw, peakYPosRaw
Writing fake all-ones data (yes, it takes that long).
Writing recommended_zchunks attribute...
[########################################] | 100% Completed | 29.1s
Virtual file virtual.h5 and list file virtual.lst successfully exported.
```

## Creation of actual processed images

...using the `proc2d.correct_image` function, which applies corrections as defined in the `YAML` file. The stack with corrected images (dask arrays, so they are not actually computed yet!) is added to the dataset.

Then, you can use the viewing widget to assess if the correction matches your expectations. If not, change the options file, and re-iterate until you're happy. (NB: The update of the widget might take slightly longer than before, as the correction is done in real time.)

Finally, run the `compute_and_save` method, which actually computes all corrected patterns and writes them to disk. Note that using the `exclude_stacks='raw_counts'` will prevent the raw data from being written into the new files. This function might take quite a while. Please follow the progress on the dask dashboard. Note that if you follow the standard workflow, this will be the first time that diffraction data is actually *written* to disk (besides the virtual one for indexing).

In [18]:

```
opts.load()
ds_compute = ds_hit # extra step just in case you want to work on a different set
img_final = proc2d.correct_image(ds_compute.raw_counts, opts,
                                 ds_compute.shots.lor_x.values,
                                 ds_compute.shots.lor_y.values,
                                 ds_compute.peak_data) # keep in mind, that this a lazy computation, so nothing is actually done yet

ds_compute.add_stack('corrected', img_final, overwrite=True, set_diff_stack=True)
```

In [19]:

```
ds_compute.view(shot=402)
```

```
sample: Lyso190304
region: 24
run: 0
crystal_id: 2
crystal_x: 692.2261410788382
crystal_y: 21.716804979253112
pos_x: 677.5261410788381
pos_y: 23.616804979253118
selected: True
subset: entry
shutter_time: 2.0
agg_len: 4
file_raw:
raw_data/LysoS2_024_00000.nxs
Event_raw: entry//53
adf1: 32.36444683953667
adf2: 12.376786337613774
center_refine_score:
0.020813135101233487
center_x: 741.7896852997765
center_y: 284.9270952168545
com_x: 741.4433189341985
com_y: 285.5343385543691
lor_hwhm: 9.424938918398581
lor_pk: 1471.0554191820365
lor_x: 741.4343634897051
lor_y: 285.9373324540116
num_peaks: 122
shift_x_mm: -40.798432691487704
shift_y_mm: -15.670990236926999
hit: True
file:
proc_data/LysoS2_024_00000_agg_hit.h
5
Event: entry//0
shot_in_subset: 0
```

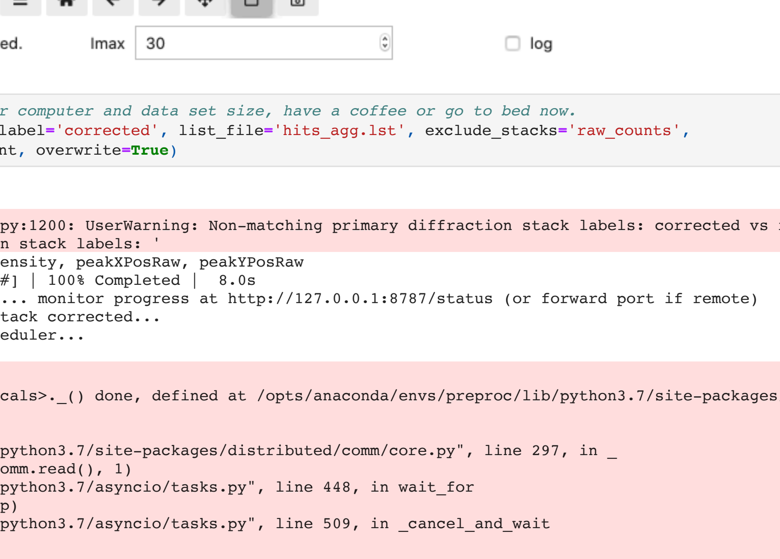

zoom rect, x=1061.21 y=382.067 [2.44]

shot 402 | selected | 1322 of 1322 shots selected. | Imax 30 | log

In [20]:

```
# run the computation. Depending on your computer and data set size, have a coffee or go to bed now.
ds_compute.compute_and_save(diff_stack_label='corrected', list_file='hits_agg.lst', exclude_stacks='raw_counts',
                            client=client, overwrite=True)
```

```
Initializing data files...
Storing meta tables...
/opts/dev/diffractem/diffractem/dataset.py:1200: UserWarning: Non-matching primary diffraction stack labels: corrected vs raw_counts
  warn(f'Non-matching primary diffraction stack labels: '
Storing meta stacks nPeaks, peakTotalIntensity, peakXPosRaw, peakYPosRaw
[########################################] | 100% Completed |  8.0s
Storing diffraction data stack corrected... monitor progress at http://127.0.0.1:8787/status (or forward port if remote)
Initializing data sets for diffraction stack corrected...
Submitting tasks to dask.distributed scheduler...
Starting computation...
Task exception was never retrieved
future: <Task finished coro=<connect.<locals>._() done, defined at /opts/anaconda/envs/preproc/lib/python3.7/site-packages/distributed/comm/core.
py:288> exception=CommClosedError()>
Traceback (most recent call last):
  File "/opts/anaconda/envs/preproc/lib/python3.7/site-packages/distributed/comm/core.py", line 297, in _
    handshake = await asyncio.wait_for(comm.read(), 1)
  File "/opts/anaconda/envs/preproc/lib/python3.7/asyncio/tasks.py", line 448, in wait_for
    await _cancel_and_wait(fut, loop=loop)
  File "/opts/anaconda/envs/preproc/lib/python3.7/asyncio/tasks.py", line 509, in _cancel_and_wait
    await waiter
concurrent.futures._base.CancelledError

The above exception was the direct cause of the following exception:

Traceback (most recent call last):
  File "/opts/anaconda/envs/preproc/lib/python3.7/site-packages/distributed/comm/core.py", line 304, in _
    raise CommClosedError() from e
distributed.comm.core.CommClosedError
```

## Broadcasting to other aggregations

With all the information we have about our initial aggregated set `ds_agg`, we can now also make other aggregations, e.g. with less or more exposure time, or including the first shot (0), which has artifacts that confuse indexing and is hence omitted.

As you will see, this is easy: make an aggregated set as before, just be cautious to make a good `file_suffix`, so you don't overwrite the existing ones. Them `merge_pattern_info` will grab all there is to know from `image_info.h5`. From there, it behaves as above: do your hit correction, compute the final image using `proc2d.correct_image`, check the outcome using `view`, and compute and save it. The peak and center data in the new files will be *exactly* as in those in `ds_agg` or e.g. the `virtual.h5` that is used for indexing. Hence, after indexing, you can use the newly made files for integration just as well - see `indexing.ipynb`.

In [36]:

```
# now, e.g. make another aggregation...
ds_0to2 = ds.aggregate(query='frame >= 0 and frame <= 2 and shutter_time == 2',
                       by=['sample', 'region', 'run', 'crystal_id'], how='sum',
                        new_folder='proc_data', file_suffix='_0to2.h5')
ds_0to2.merge_pattern_info('image_info.h5')
ds_0to2_hit = ds_0to2.get_selection(f'num_peaks > {opts.min_peaks}', file_suffix='_hit.h5')
```

```
Monotonous aggregation: True
File/subset remixing: False
Frame aggregation: True
Acq. run aggregation: False
Discarding shot table columns: ['Event', 'frame', 'shot_in_subset']
Persisting stacks to memory:
Single-file dataset, disabling parallel I/O.
No feature list in data set ('/%/map/features not found in image_info.h5.'). That's ok if it's a virtual or info file.
Persisting stacks to memory: nPeaks, peakTotalIntensity, peakXPosRaw, peakYPosRaw
Persisting stacks to memory: nPeaks, peakXPosRaw, peakYPosRaw, peakTotalIntensity
1322 shots out of 2146 selected.
Persisting stacks to memory: nPeaks, peakXPosRaw, peakYPosRaw, peakTotalIntensity
```

In [39]:

```
# ...and correct the images
opts.load()
ds_compute = ds_0to2_hit
img_final = proc2d.correct_image(ds_compute.raw_counts, opts,
                                 ds_compute.shots.lor_x.values,
                                 ds_compute.shots.lor_y.values,
                                 ds_compute.peak_data) # keep in mind, that this a lazy computation, so nothing is actually done yet

ds_compute.add_stack('corrected', img_final, overwrite=True, set_diff_stack=True)
ds_compute.view()
```

```
sample: Lyso190304
region: 19
run: 0
crystal_id: 24
crystal_x: 166.52472858866105
crystal_y: 788.826296743064
pos_x: 144.92472858866105
pos_y: 791.426296743064
selected: True
subset: entry
shutter_time: 2.0
agg_len: 3
file_raw:
raw_data/LysoS2_019_00000.nxs
Event_raw: entry//612
adf1: 29.908668998024442
adf2: 10.678603693548705
center_refine_score:
0.006549543119855038
center_x: 761.1423528657535
center_y: 248.70339135217256
com_x: 760.4894094539833
com_y: 250.42694165143274
lor_hwhm: 11.321482035002811
lor_pk: 1035.861406920318
lor_x: 760.4112782960242
lor_y: 249.70202594439667
num_peaks: 123
shift_x_mm: -41.862829407616445
shift_y_mm: -13.67868652436949
ii_from: 602
file:
proc_data/LysoS2_019_00000_0to2_hit.
h5
Event: entry//7
shot_in_subset: 7
```

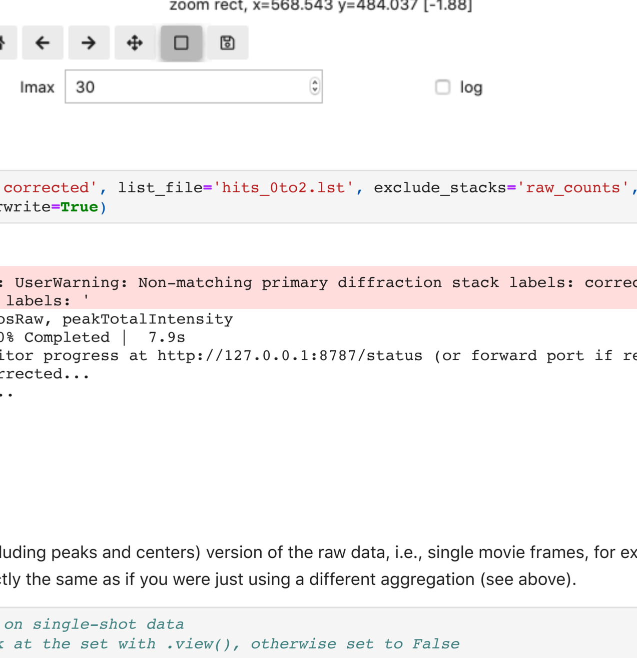

zoom rect, x=568.543 y=484.037 [-1.88]

shot 296 | selected | 1322 of 1322 shots selected. | Imax 30 | log

Now, `ds_0to2_hit` has everything to be written to disk!

In [41]:

```
ds_0to2_hit.compute_and_save(diff_stack_label='corrected', list_file='hits_0to2.lst', exclude_stacks='raw_counts',
                             client=client, overwrite=True)
```

```
Initializing data files...
Storing meta tables...
/opts/dev/diffractem/diffractem/dataset.py:1200: UserWarning: Non-matching primary diffraction stack labels: corrected vs raw_counts
  warn(f'Non-matching primary diffraction stack labels: '
Storing meta stacks nPeaks, peakXPosRaw, peakYPosRaw, peakTotalIntensity
[########################################] | 100% Completed |  7.9s
Storing diffraction data stack corrected... monitor progress at http://127.0.0.1:8787/status (or forward port if remote)
Initializing data sets for diffraction stack corrected...
Submitting tasks to dask.distributed scheduler...
Starting computation...
```

## Broadcasting to single shots

(Advanced)

Now, we want to make a corrected and annotated (i.e., including peaks and centers) version of the raw data, i.e., single movie frames, for example to study radiation damage or be flexible during merging. This is done essentially exactly the same as if you were just using a different aggregation (see above).

In [ ]:

```
# now, do exactly the same thing as above, but on single-shot data
unchunk = True # IMPORTANT: set to True to look at the set with .view(), otherwise set to False

ds_sgl = ds.get_selection('frame >= 0 and frame < 10', file_suffix='_allframe.h5', new_folder='proc_data')

ds_sgl.merge_pattern_info('image_info.h5')
ds_sgl = ds_sgl.get_selection(f'num_peaks > {opts.min_peaks}', file_suffix='_hit.h5')

if unchunk:
    ds_sgl.rechunk_stacks(1)

opts.load()
ds_compute = ds_sgl
img_final = proc2d.correct_image(ds_compute.raw_counts, opts,
                                 ds_compute.shots.lor_x.values,
                                 ds_compute.shots.lor_y.values,
                                 ds_compute.peak_data) # keep in mind, that this a lazy computation, so nothing is actually done yet

ds_compute.add_stack('corrected', img_final, overwrite=True, set_diff_stack=True)
```

In [ ]:

```
# ...and run the computation
ds_compute.compute_and_save(diff_stack_label='corrected', list_file='hits-allframe.lst', exclude_stacks='raw_counts',
                            client=client, overwrite=True)
```

## Make cumulative-sum files

(Advanced)

Finally, you can also create a set of files, which instead of single frames, have their cumulative sums, which means that you can pick in hindsight which ones you want to use for the later steps. Some might prefer a workflow where you just make files for different aggregations (as above) that you think make sense.

Anyway - for this case, the function `transform_stack_group` does exactly what you want: a cumulative sum over each group in your stack matching one unique crystal. The rest is as usual.

In [ ]:

```
# only if restarting from here
ds_sgl = Dataset.from_files('hits-allframe.lst', chunking=20)
```

In [ ]:

```
# copy data set and apply transform function, which defaults to cumulation
ds_cum_0 = ds_sgl.get_selection('True', file_suffix='_cum_from_0.h5')
ds_cum_0.transform_stack_groups(stacks='corrected')
```

In [ ]:

```
# more sophisticated: start cumulation only at second frame
# this reduces the signal somewhat and throws away the (maybe) most undamaged
# frame, but avoids streak artifacts that might be in it
cum_from_1 = lambda x: np.concatenate([x[:1,...], np.cumsum(x[1:,...], axis=0)], axis=0)
ds_cum_1 = ds_sgl.get_selection('True', file_suffix='_cum_from_1.h5')
ds_cum_1.transform_stack_groups(stacks='corrected', func=cum_from_1)
```

In [ ]:

```
%matplotlib widget
ds_cum_1.view()
```

In [ ]:

```
# run the computation. Depending on your computer and data set size, have a coffee or go to bed now.
ds_cum_0.compute_and_save(diff_stack_label='corrected', list_file='hits_cum-0.lst', exclude_stacks='raw_counts',
                          client=client, overwrite=True)
ds_cum_1.compute_and_save(diff_stack_label='corrected', list_file='hits_cum-1.lst', exclude_stacks='raw_counts',
                          client=client, overwrite=True)
```
