# Supplementary material for: Serial Electron Diffraction Data Processing With diffractem and CrystFEL
Source: Front Mol Biosci. 2021 May 19;8:624264. doi: 10.3389/fmolb.2021.624264 (PMC8171297; doi:10.3389/fmolb.2021.624264)

In [ ]:

```
# only for development
%load_ext autoreload
%autoreload 2
```

In [1]:

```
import warnings
warnings.filterwarnings('ignore')
import matplotlib.pyplot as plt
from diffractem import io, tools
from diffractem.stream_parser import StreamParser, make_substream
import numpy as np
import pandas as pd
import os
import matplotlib
import seaborn as sns
bin_path = '/opts/crystfel_master/bin/' # might be different than standard
from glob import glob
from concurrent.futures import ProcessPoolExecutor, ThreadPoolExecutor
```

Merging and first validation of serial data sets

...from stream files, mostly using `partialator`, `ambigator`, `check_hkl` and `compare_hkl` from CrystFEL, plus some nice plotting functions. Handles parallel processing of merging runs with different parameters and/or input stream files, as well as creation of custom-split files.

Contains the following parts:

- Pre-processing of stream files, e.g. for making sub-streams by random sampling, or preparing split-lists
- Generation of a command script for partilator, which runs it with a bunch of different settings, either directly in a shell, or by submission to a SLURM queue
- Batch analysis of `hkl` files using CrystFEL's tools (in parallel) and results parsing
- Plotting of results as function of resolution shell and crystal number

Step 1: Polishing stream-files

...which contain the indexing solutions and integrated intensities from each shot. Here, you might e.g. want to generate a file for `custom-split` (see `man partialator`), or to generate stream files with random subsets of events. If non-random subsets (e.g. the first N crystals) are also ok, you can use `partialator`'s `start-after` and `stop-after` options instead!

If you don't care about any of this, you can skip this section and proceed to Step 2

We start by loading the stream file...

In [2]:

```
stream = StreamParser('streams/0to2.stream')
print(f'Stream file contains {stream.num_crystals} crystals in {stream.num_shots} shots')
```

```
Stream file contains 1152 crystals in 1322 shots
```

Random sub-sampling

Creates stream-files with *random* subsets of shots and suffix `-N_<# of crystals>.stream`.

In [ ]:

```
subset_range = list(range(200, stream.num_crystals, 200)) + [stream.num_crystals]

with ThreadPoolExecutor() as exc:
    Ntotal = (stream.shots.indexed_by != 'none').sum()
    exc.map(lambda N: make_substream(stream, N,
                                     filename=stream.filename.rsplit('.',1)[0] + f'-N_{N}.stream',
        query='indexed_by != "none"'), subset_range)
```

Custom-split file

Creates a custom-split file for the stream from some parameter in `stream.shots`. This can be very useful e.g. if you want to run a stream containing many time-points (or aggregations) of the same crystals, which you want to scale consistently (example 1). For many other interesting applications, you may have to bin the parameters first, though. This is shown here for the example of `profile_radius`, for which we will do a quantile-based binning (example 2).

In [ ]:

```
# EXAMPLE 1: dose-fractionation frames.
# only works if the stream file is from a multi-frame dataset
split_data = stream.shots[['file', 'Event', 'hdf5/%/shots/frame']]
split_data.to_csv(stream.filename.rsplit('.', 1)[0] + '_split.txt', index=False, header=False, sep=' ')
```

In [3]:

```
# EXAMPLE 2: profile radius
n_quantiles = 4 # number of quantile groups.

pr = stream.shots.profile_radius.str.split(expand=True)[0].astype(float)
group = (pd.qcut(pr, n_quantiles, labels=False)+1).fillna(0).astype(int)
split_data = pd.concat([stream.shots[['file', 'Event']], group], axis=1)
split_data.to_csv(stream.filename.rsplit('.', 1)[0] + '_split.txt', index=False, header=False, sep=' ')
```

Step 2: Preparation of a partialator script

...which can run partialator with a range of different settings, in order to find the one that fits your data best. The partialator parameters are all set in the `popts` dictionary. Please consult the parameter descriptions available via `man partialator`. If you put a list in any of them, `partialator` will be run in all possible combinations. In this example we use `stop-after` to get a range of different crystal numbers (we might as well have used the random sampling from streams as shown above) and test two merging models.

It comes in different flavors - either for direct execution, or for submission to a SLURM queue, depending on what you set for the `slurm` argument*. If you want a custom-split, set `split=True` and create a split list file with the same name and path as the stream file and ending `_split.txt` - see examples above how to make it. We will however not work with this for now.

`call_partialator` will create a script file `partialator_run.sh` and return a `DataFrame` containing a list of the settings with which partialator is run, and what the filename will be.

*(Note that, unlike with `call_indexamajig_slurm`, no further options are available to e.g. make an archive file for transfer. Just copy the script and your stream file(s) to the cluster)

In [4]:

```
# get a list of stream files
stream_list = glob('streams/*.stream')

popts = {'no-polarisation': True, 'no-Bscale': False, 'no-scale': False,
        'force-bandwidth': 2e-5,  'force-radius': False, 'force-lambda': 0.0251,
            'push-res': 1.4,  'min-measurements': [3, ], 'model': ['unity', 'xsphere'],
            'symmetry': '422', 'stop-after': list(range(200, 1147, 200)) + [1147],
            'no-logs': False, 'iterations': 3, 'j': 10}

# you need to set those if you want to use slurm to submit merging runs
slurm_opts = {'C': 'scratch',
                'partition': 'medium',
                'time': '"04:00:00"',
                'nodes': 1}

tools.call_partialator(stream_list, popts, par_runs=4,
                       split=False, out_dir='merged',
                       slurm=False, cache_streams=False,
                       slurm_opts=slurm_opts)
```

```
Please run partialator_run.sh to start merging.
```

Out[4]:

	input	min_measurements	model	stop_after	hklfile
0	streams/0to2.stream	3	unity	200	merged/0to2__3__unity__200.hkl
1	streams/0to2.stream	3	unity	400	merged/0to2__3__unity__400.hkl
2	streams/0to2.stream	3	unity	600	merged/0to2__3__unity__600.hkl
3	streams/0to2.stream	3	unity	800	merged/0to2__3__unity__800.hkl
4	streams/0to2.stream	3	unity	1000	merged/0to2__3__unity__1000.hkl
...	...	...	...	...	...
79	streams/0to8.stream	3	xsphere	400	merged/0to8__3__xsphere__400.hkl
80	streams/0to8.stream	3	xsphere	600	merged/0to8__3__xsphere__600.hkl
81	streams/0to8.stream	3	xsphere	800	merged/0to8__3__xsphere__800.hkl
82	streams/0to8.stream	3	xsphere	1000	merged/0to8__3__xsphere__1000.hkl
83	streams/0to8.stream	3	xsphere	1147	merged/0to8__3__xsphere__1147.hkl

84 rows × 5 columns

In [ ]:

```
# example how to send data to a cluster
# !scp -r streams rbueckel@transfer.gwdg.de:~/SHARED/EDIFF/temp
# !scp partialator_run.sh rbueckel@transfer.gwdg.de:~/SHARED/EDIFF/temp
```

In [ ]:

```
# example how to get it back from a cluster
# %mkdir merged
# !scp 'rbueckel@transfer.gwdg.de:~/SHARED/EDIFF/temp/merged/*.hkl*' merged/
```

Step 3: Analyze and validate results

...assuming your partialator run has finished, and using CrystFELs `check_hkl` and `compare_hkl` tools. The results are parsed into *pandas* DataFrames using `tools.analzye_hkl` and automatically labeled. While `overall` contains the overall figures of merit for all data frames, `sd` is a flat list of all per-shell and per-setting FOMs. You can use pandas' `groupby` and `pivot` features to get from this table whatever you need conveniently.

Finally, plots of figures of merit vs. resolution shell can be generated.

Parse available files

If you haven't just run the merging before, but started from here, you need a `settings` DataFrame to get started. This cell does that for you, mostly rather accurately, using `tools.get_hkl_settings`. It can then also be used to reject some settings from further analysis (not needed now, but examples are in comments), or mangle with the data columns to have more convenient indicators (done here)

In [5]:

```
# check what hkls we have available....
settings = tools.get_hkl_settings('merged/*.hkl', unique_only=True, custom_split=True)

# reject some for analysis...
# settings = settings.query('model != "random"')
# settings = settings.loc[settings.input.str.contains('v_2')]
# settings.drop(columns='input', inplace=True)

# or do some name mangling... (here: make sure that the folder is stripped of the stream)
settings['input'] = settings['input'].str.rsplit('/', 1, expand=True).iloc[:,-1]
```

Run the analysis

This is done using the `tools.analyze_hkl` function. However, as the analysis is single-threaded, we can get much faster by doing it in parallel using a `ProcessPoolExecutor` (see the documentation of the `concurrent.futures` package if you want to know more. Concise tables of the results are written into the `shell/` subdirectory.

(if there is trouble with finding the CrystFEL executables, set the bin_path parameter manually)

In [6]:

```
default_symmetry = '422'
highres = 1.75 # highest shell, in A
nshells = 10

# tools.analyze_hkl() #...is used using ProcessPoolExecutor

ftrs = {}
with ProcessPoolExecutor() as exc:
    for _, s in settings.iterrows():
        ftrs[s.hklfile] = exc.submit(tools.analyze_hkl, fn=s.hklfile, cell='gencell.cell',
                                     point_group=s.symmetry if 'symmetry' in s else default_symmetry,
                                     highres=highres, nshells=nshells, bin_path='/opts/crystfel_master/bin')

err = {lbl: v.exception() for lbl, v in ftrs.items() if v.exception()}
if err:
    print('Analysis gave errors!', str(err))
out = {lbl: v.result() for lbl, v in ftrs.items() if not v.exception()}

sd = pd.concat([v.result()[0].assign(hklfile=lbl)
                for lbl, v in ftrs.items()
                    if not v.exception()], axis=0).merge(
    settings, on='hklfile')

overall = pd.concat([pd.DataFrame(v.result()[1], index=[lbl])
                for lbl, v in ftrs.items()
                     if not v.exception()], axis=0).merge(
    settings, right_on='hklfile', left_index=True).rename(
    columns={'<snr>': 'SNR', 'redundancy': 'Red', 'completeness': 'Compl', 'CC*': 'CCstar'})

# write out results
%rm -f shell/*
for ident, grp in sd.groupby(['hklfile']):
    grp.sort_values('Center 1/nm')[['Center 1/nm', 'nref', 'Possible', 'Compl', 'Meas', 'Red', 'SNR',
          'Std dev', 'Mean', 'd/A', 'Min 1/nm', 'Max 1/nm', 'CC', 'CCstar',
          'Rsplit']].to_csv(f'shell/{ident.rsplit("/",1)[-1]}.csv', index=False, float_format='%.2f')
```

Example to show results

...using the result DataFrame's `pivot` function.

In [7]:

```
# convenient function to get FOMs. Set the one you want as 'value'
sd.pivot(index='d/A', columns='hklfile', values=['CC']).sort_index(ascending=False)
```

Out[7]:

hklfile<br>d/A	merged/0to1__3__unity__1000.hkl	merged/0to1__3__unity__1147.hkl	merged/0to1__3__unity__200.hkl	merged/0to1__3__unity_
6.80	0.891341	0.899728	0.662385	[illegible]
3.34	0.883699	0.889219	0.671010	[illegible]
2.79	0.802455	0.811093	0.643565	[illegible]
2.49	0.714571	0.719277	0.540547	[illegible]
2.29	0.710093	0.707172	0.397646	[illegible]
2.14	0.560271	0.567976	0.249249	[illegible]
2.02	0.438179	0.484880	0.226432	[illegible]
1.93	0.342366	0.385313	0.433509	[illegible]
1.85	0.218812	0.243168	-1.000000	[illegible]
1.78	0.146146	0.096615	NaN	[illegible]

10 rows × 84 columns

Step 4: Analysis Plots

...ideally using an interactive backend, like `qt`, or `widget`. You can choose which 4 FOMs to plot as a function of resolution, and their display ranges. From the $CC = 1/7$, or $CC^* = 1/2$ criterion (which are automatically drawn if you ask for those), we can read off a reasonable cut-off for resolution. To learn about what FOMs you can plot, please look at `sd.columns`. The `sdsel=sd.query(......)` allows you to only plot a sub-selection - e.g. here only the full set with unity merging, but all aggregations.

Fig 1: Figures of Merit vs resolution shell

You can pick subsets of merging runs using `sd.query`, and set the x axis by setting `angstrom`.

In [10]:

```
%matplotlib widget
# SETTINGS ---

fh, axs = plt.subplots(2, 2, figsize=(18/2.54,15/2.54), dpi=120, sharex=True)
lsp, lrow = 0.85, 4 # space near top left for legend, and # of legend columns

# pick your FOMs and their y ranges
FOMs = [('CC', 0, 1), ('Mean', 0, 100), ('Compl', 0, 100), ('Red', 0, 100)]
sdsel = sd.query('stop_after > 1000 and model=="xsphere"')
angstrom = False # if True, show x axis in A, instead of 1/nm

# ------

try:
    import seaborn as sns
    sns.set('notebook','whitegrid') # optional. Set a style...
except:
    print('Seaborn not installed, it seems.')

axs = axs.ravel()

# ids = get_id_table(sdsel['identifier'])
idcols = [cn for cn, col in sdsel[settings.columns].iteritems()
          if len(col.unique()) > 1 and (cn != 'hklfile')]
print('Legend is', ' '.join(idcols))

for ident, grp in sdsel.groupby(['hklfile']):

    ls = '-'

    lbl = tuple(grp[idcols].drop_duplicates().values.astype(str).ravel())

    for ax, (fom, ymin, ymax) in zip(axs, FOMs):
        ax.plot(grp['d/A'] if angstrom else grp['Center 1/nm'], grp[fom],
                label=' '.join(lbl), ls=ls)
        ax.set_title(fom)
        ax.set_ylim((ymin, ymax))
        if angstrom:
            ax.set_xlim(sorted(ax.get_xlim(), reverse=True))
        ax.grid(True)
        if fom in ['CC', 'CCstar']:
            ax.axhline(0.143 if fom == 'CC' else 0.5,ls=':')

lg = fh.legend(*ax.get_legend_handles_labels(), ncol=lrow,
               fontsize='xx-small', loc='lower center',
               bbox_to_anchor=(0.5, lsp), frameon=True)
axs[-1].set_xlabel(r'Resolution shell/Å' if angstrom else r'Resolution shell/nm$^{-1}$')
plt.draw()
# lpos = lg.get_window_extent()

fh.subplots_adjust(wspace=0.3, top=lsp-0.05)
```

```
Legend is input
```

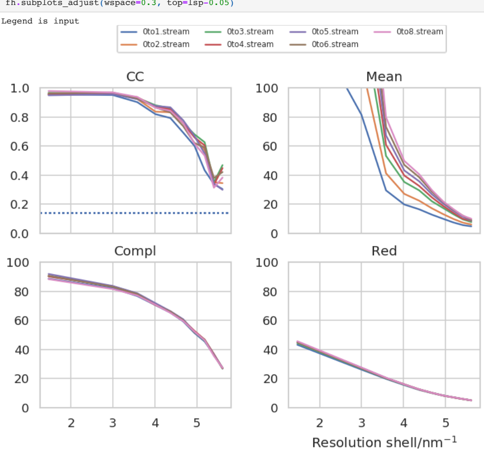

Fig. 2: overall and single-shell FOM vs. crystal number.

Obviously only makes sense for data sets which have multiple crystal numbers. If you did not define this using `stop_after` or `start_after`, you'll have to mangle around with e.g. hklfile names yourself before. The plots display values of a selected shell in dashed lines, which you can pick in the variable `res`. The available values are printed when running the cell.

In [11]:

```
# SETTINGS ---

N_col = 'stop_after' # column containing the number of crystals

fh, axs = plt.subplots(2,2,figsize=(18/2.54,15/2.54),dpi=120,sharex=True)

# pick your FOMs and their y ranges
FOMs = [('CC', 0, 1), ('Compl', 0, 100), ('Rsplit', 0, 100), ('Red', 0, 50)]

# resolution bin for dashed plot
res = 1.93
ovsel = overall.query('input in ["0to1.stream", "0to2.stream", "0to8.stream"]')

# -----

try:
    import seaborn as sns
    sns.set('notebook','whitegrid') # optional. Set a style...
except:
    print('Seaborn not installed, it seems.')

axs = axs.ravel()

idcols = [cn for cn, col in sd[settings.columns].iteritems()
          if len(col.unique()) > 1 and (cn not in  ['hklfile', N_col])]

print('Available resolution bins are', ' '.join(sd['d/A'].unique().astype(str)))
print('Legend is', ' '.join(idcols))

for idcol, grp in ovsel.groupby(idcols if len(idcols) else np.ones(len(ovsel))):

    sdsel = sd.merge(grp, on='hklfile', validate='m:1', suffixes=('','_ov'))
    sdsel = sdsel[sdsel['d/A'] == res].sort_values(N_col)

    grp = grp.sort_values(N_col)
    lbl = tuple(grp[idcols].drop_duplicates().values.astype(str).ravel())
    for ax, (fom, xmin, xmax) in zip(axs, FOMs):
        ph = ax.plot(grp[N_col], grp[fom], label=' '.join(lbl), ls='-')
        ax.plot(sdsel[N_col], sdsel[fom], color=ph[0].get_color(), ls='--')
        ax.set_title(fom)
        ax.set_ylim((xmin, xmax))
        ax.grid(True)
        if fom in ['CC', 'CCstar']:
            ax.axhline(0.143 if fom == 'CC' else 0.5,ls=':')

axs[-1].legend(ncol=2, fontsize='xx-small')
axs[-1].set_xlabel(r'Crystal number')

fh.subplots_adjust(wspace=0.3)
```

```
Available resolution bins are 6.8 3.34 2.79 2.49 2.29 2.14 2.02 1.93 1.85 1.78
Legend is input model
```

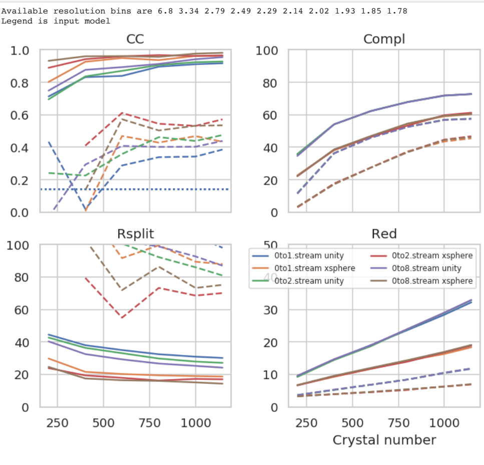

Step 5: publication-ready plots

Another plot section, which is more dataset-specific and less convenient, but really makes good plots for final reports with slightly more effort. Needs to be adapted to each dataset manually, so you'll have to do that if you copied this notebook from another dataset.

This is *not* part of a general workflow tutorial - most likely it will not work without lots of customization

Versus Resolution shell

In [13]:

```
import matplotlib as mpl

mpl.rc('axes', titleweight='normal', labelweight='normal')

# Dataset-separated FOM vs resolution plot
import seaborn as sns
sns.set('paper','whitegrid') # optional. Set a style...

FOMs = [(['CC', None], 0, 1, '$CC_{1/2}$'),
        (['Compl', None], 0, 100, 'Completeness'),
       (['Red', None], 0, 50, 'Redundancy')]#, R_\mathrm{work}$ (dashed)')]

# select the data subset to show. Add "cmp" to the method list if you want relative data
sd_sel = sd.query(f'stop_after > 1000 and input in ["0to1.stream", "0to2.stream", "0to8.stream"]')

plt.close('all')

fh, axs= plt.subplots(len(FOMs), 1, figsize=(8.5/2.54, 15/2.54), sharex=True, dpi=72,
                      gridspec_kw={'hspace':0.1, 'wspace':0.1, 'height_ratios': [1]*len(FOMs)})

# The 'zzz' rename is required to put the plots in proper order
last_model = ''
for (model, agg, hklfile), grp in \
    sd_sel.groupby(['model', 'input', 'hklfile'], sort=True):

    for ii, (fom, ymin, ymax, cpt) in enumerate(FOMs):
        ax = axs[ii]

        if model != last_model:
            ax.set_prop_cycle(None)

        if fom[0] == 'CC':
            ax.axhline(0.143, color='k', alpha=0.1)

        lbl = f'{2*int(agg[3])+1} ms' if model=='unity' else ''
        src = grp
        ph = ax.plot(src['Center 1/nm'], src[fom[0]],
                     label=lbl,
                     ls='-' if model=='unity' else '--',
                     marker='o' if model=='unity' else 'v', markersize=3,
                     fillstyle='none')
        c = [p.get_color() for p in ph]
        if fom[1] is not None:
            lbl = None
            ax.plot(src['Center 1/nm'], src[fom[1]],
                    label=lbl, color=c[0], ls='--',
                    marker='o' if model=='unity' else 'v', fillstyle='none')

        # common axis settings
        ax.set_ylim((ymin, ymax))
        ax.set_xticks([10/d for d in range(6,1,-1)])
        ax.grid(True)

        # stuff appearing for specific axes only
        if ii == 0:
            ax.legend()
        ax.set_ylabel(cpt)
        if ii == (len(FOMs)-1):
            ax.set_xlabel('Resolution (Å)')
            ax.set_xticklabels([f'{(10/float(l)):.0f}' for l in ax.get_xticks()])
        else:
            ax.set_xticklabels([])

    last_model = model

ax.set_xlim(10/4.5, ax.get_xlim()[1])

plt.savefig(f'fom_vs_res.pdf', transparent=True, bbox_inches='tight')
```

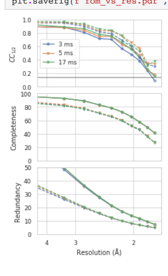

Versus Crystal Number

In [14]:

```
# PART 2: FOMs at fixed resolution/overall vs. crystal number
# %matplotlib inline
# MAIN TEXT
target = 'main'
FOMs = [(['CC_ov', 'CC'], 0, 1, '$CC_{1/2}$'),
        (['Compl_ov', 'Compl', None], 0, 100, 'Completeness'),
       (['Red_ov', 'Red'], 0, 50, 'Redundancy')]

# make subset (including carving out the highest shell only)
hishell = 1.85
sd_sel = sd.loc[sd['d/A'] == hishell,:].sort_index() # xfel9 / ssx9: 0.9.0

# merge overall data. Inner merge, so it is sub-selected automatically
sd_sel = sd_sel.merge(overall, on=['model', 'input', 'stop_after'],
                      how='inner', suffixes=('', '_ov'))

# select crystal number range
sd_sel = sd_sel.query('input == "0to2.stream"')

fh, axs= plt.subplots(len(FOMs), 1, figsize=(8.5/2.54, 15/2.54), sharex=True, dpi=72,
                      gridspec_kw={'hspace':0.1, 'wspace':0.1, 'height_ratios': [1]*len(FOMs)})

for (model, agg), grp in sd_sel.groupby(['model', 'input'], sort=True):
    grps = grp.sort_values(by='stop_after').rename(columns={'stop_after': 'crystals'})

    for ii, (fom, ymin, ymax, cpt) in enumerate(FOMs):
        ax = axs[ii]
        if fom[0] in grps.columns:
            ph = ax.plot(grps.crystals, grps[fom[0]],
                     label=f'{model}',
                     linestyle='-', marker='o' if model=='unity' else 'v',
                        fillstyle='none', markersize=3)
        if (fom[1] is not None) and (fom[1] in grps.columns):
            ax.plot(grps.crystals, grps[fom[1]],
    #                label=f'{method.upper()} ({fom[1]})',
                    label = None,
                    color=ph[0].get_color(),
                    linestyle='--', marker='o' if model=='unity' else 'v',
                    fillstyle='none', markersize=3)

        ax.set_ylim((ymin, ymax))
        ax.set_ylabel(cpt)

        if (ii == 0):
            ax.legend()
        ax.set_xlim(100, 1300)
        if ii == (len(FOMs)-1):
            ax.set_xlabel('Crystal number')
plt.savefig(f'fom_vs_number.pdf', transparent=True, bbox_inches='tight')
```

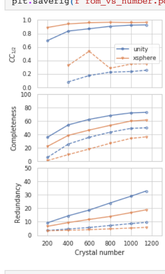

In [15]:

```
import matplotlib as mpl

mpl.rc('axes', titleweight='normal', labelweight='normal')
plt.close('all')

# Dataset-separated FOM vs resolution plot
import seaborn as sns
sns.set('paper','whitegrid') # optional. Set a style...

FOMs = [(['CC', None], 0, 1, '$CC_{1/2}$'),
        (['Compl', None], 0, 100, 'Completeness'),
       (['Red', None], 0, 50, 'Redundancy')]#, R_\mathrm{work}$ (dashed)')]


fh, axs_both = plt.subplots(len(FOMs), 2, figsize=(17/2.54, 15/2.54), sharex=False, sharey=False, dpi=7
                      gridspec_kw={'hspace':0.1, 'wspace':0.1, 'height_ratios': [1]*len(FOMs)})

# select the data subset to show. Add "cmp" to the method list if you want relative data
sd_sel = sd.query(f'stop_after > 1000 and input in ["0to1.stream", "0to2.stream", "0to8.stream"]')

axs = axs_both[:, 0]

# The 'zzz' rename is required to put the plots in proper order
last_model = ''
for (model, agg, hklfile), grp in \
    sd_sel.groupby(['model', 'input', 'hklfile'], sort=True):

    for ii, (fom, ymin, ymax, cpt) in enumerate(FOMs):
        ax = axs[ii]

        if model != last_model:
#            print(last_model, model)
            ax.set_prop_cycle(None)

        if fom[0] == 'CC':
            ax.axhline(0.143, color='k', alpha=0.1)

        lbl = f'{2*int(agg[3])+1} ms' if model=='unity' else ''
#        lbl = f'{model}, {2*int(agg[3])+1} ms'
        src = grp
        ph = ax.plot(src['Center 1/nm'], src[fom[0]],
                     label=lbl,
                     ls='-' if model=='unity' else '--',
                     marker='o' if model=='unity' else 'v', markersize=3,
                     fillstyle='none')
        c = [p.get_color() for p in ph]
        if fom[1] is not None:
            lbl = None
            ax.plot(src['Center 1/nm'], src[fom[1]],
                    label=lbl, color=c[0], ls='--',
                    marker='o' if model=='unity' else 'v', fillstyle='none')

        # common axis settings
        ax.set_ylim((ymin, ymax))
        ax.set_xticks([10/d for d in range(6,1,-1)])
        ax.grid(True)

        # stuff appearing for specific axes only
        if ii == 0:
#            ax.set_title('MB ($N=8000$)' if sample=='mb' else 'FAcD ($N=10000$)')
            ax.legend(ncol=1)
        ax.set_ylabel(cpt)
        if ii == (len(FOMs)-1):
            ax.set_xlabel('Resolution (Å)')
            ax.set_xticklabels([f'{(10/float(l)):.0f}' for l in ax.get_xticks()])
        else:
            ax.set_xticklabels([])

        ax.set_xlim(10/4.5, ax.get_xlim()[1])

    last_model = model

# plt.savefig(f'fom_vs_res.pdf', transparent=True, bbox_inches='tight')

# PART 2: FOMs at fixed resolution/overall vs. crystal number
# %matplotlib inline
# MAIN TEXT
target = 'main'
FOMs = [(['CC_ov', 'CC'], 0, 1, '$CC_{1/2}$'),
        (['Compl_ov', 'Compl', None], 0, 100, 'Completeness'),
       (['Red_ov', 'Red'], 0, 50, 'Redundancy')]

# make subset (including carving out the highest shell only)
hishell = 1.85
sd_sel = sd.loc[sd['d/A'] == hishell,:].sort_index() # xfel9 / ssx9: 0.9.0

# merge overall data. Inner merge, so it is sub-selected automatically
sd_sel = sd_sel.merge(overall, on=['model', 'input', 'stop_after'],
                      how='inner', suffixes=('', '_ov'))

# select crystal number range
sd_sel = sd_sel.query('input == "0to2.stream"')

axs = axs_both[:, 1]

for (model, agg), grp in sd_sel.groupby(['model', 'input'], sort=True):
    grps = grp.sort_values(by='stop_after').rename(columns={'stop_after': 'crystals'})

    for ii, (fom, ymin, ymax, cpt) in enumerate(FOMs):
        ax = axs[ii]
        if fom[0] in grps.columns:
            ph = ax.plot(grps.crystals, grps[fom[0]],
                     label=f'{model}',
                     linestyle='-', marker='o' if model=='unity' else 'v',
                        fillstyle='none', markersize=3)
        if (fom[1] is not None) and (fom[1] in grps.columns):
            ax.plot(grps.crystals, grps[fom[1]],
    #                label=f'{method.upper()} ({fom[1]})',
                    label = None,
                    color=ph[0].get_color(),
                    linestyle=':', marker='o' if model=='unity' else 'v',
                    fillstyle='none', markersize=3)

        ax.set_ylim((ymin, ymax))
#         ax.set_ylabel(cpt)
        ax.set_yticklabels([])

        if (ii == 0):
            ax.legend()
        ax.set_xlim(100, 1300)
        if ii == (len(FOMs)-1):
            ax.set_xlabel('Crystal number')
        else:
            ax.set_xticklabels([])

plt.savefig(f'fom_all.pdf', transparent=True, bbox_inches='tight')
```

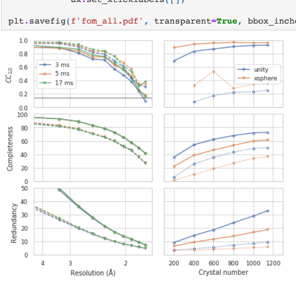

Supplement: Supplementary file 3 [file Presentation2.PDF]
